# Microorganisms associated with *Sporobolus anglicus*, an invasive dimethylsulfoniopropionate producing salt marsh plant, are an unrecognized sink for dimethylsulfide

**DOI:** 10.3389/fmicb.2022.950460

**Published:** 2022-09-29

**Authors:** Eileen Kröber, Anna Mankowski, Hendrik Schäfer

**Affiliations:** ^1^School of Life Sciences, Gibbet Hill Campus, University of Warwick, Coventry, United Kingdom; ^2^Max Planck Institute for Marine Microbiology, Bremen, Germany

**Keywords:** dimethylsulfide, salt marsh, *Methylophaga*, stable isotope probing, *Sporobolus anglicus*

## Abstract

**Background:**

Saltmarshes are hotspots of organosulfur compound cycling due to production of dimethylsulfoniopropionate (DMSP) by benthic microorganisms, macroalgae, and saltmarsh vegetation. Degradation of DMSP is a source of dimethylsulfide (DMS), an important precursor for formation of secondary organic aerosol. Microorganisms degrading DMS play a role in controlling the amount of DMS available for emission into the atmosphere. Previous work has implicated sediment microbial populations as a major sink for DMS. Here, we show that *Sporobolus anglicus* (previously known as *Spartina anglica*), a widely distributed saltmarsh plant, is colonized by DMS-degrading microorganisms.

**Methods:**

Dimethylsulfide degradation potential was assessed by gas chromatography and ^13^C-DMS stable isotope probing, microbial community diversity and functional genetic potential in phyllosphere and rhizosphere samples was assessed by high-throughput sequencing of 16S rRNA gene amplicons, cloning and sequencing of methanethiol oxidase genes, and by metagenomic analysis of phyllosphere microbial communities.

**Results:**

The DMS degradation potential of microbial communities recovered from phyllosphere and rhizosphere samples was similar. Active DMS-degraders were identified by ^13^C-DMS stable isotope probing and included populations related to *Methylophaga* and other *Piscirickettsiaceae* in rhizosphere samples. DMS-degraders in the phyllosphere included *Xanthomonadaceae* and *Halothiobacillaceae*. The diversity in sediment samples of the methanethiol oxidase (*mtoX*) gene, a marker for metabolism of methanethiol during DMS and DMSP degradation, was similar to previously detected saltmarsh *mtoX*, including those of *Methylophaga* and *Methylococcaeae*. Phyllosphere *mtoX* genes were distinct from sediment *mtoX* and did not include close relatives of cultivated bacteria. Microbial diversity in the phyllosphere of *S. anglicus* was distinct compared to those of model plants such as rice, soybean, clover and *Arabidopsis* and showed a dominance of *Gammaproteobacteria* rather than *Alphaproteobacteria*.

**Conclusion:**

The potential for microbial DMS degradation in the phyllosphere and rhizosphere of *Sporobolus anglicus* suggest that DMS cycling in saltmarshes is more complex than previously recognised and calls for a more detailed assessment of how aboveground activities affect fluxes of DMS.

## Background

Dimethylsulfide (DMS) is the most abundant biologically formed sulfur compound emitted to the atmosphere and performs a crucial role in the global sulfur cycle (Lovelock et al., 1972). As an important precursor for secondary atmospheric aerosols DMS affects atmospheric chemistry and contributes to transport of sulfur from the marine environment to terrestrial environments via the atmosphere (Charlson et al., 1987; Carslaw et al., 2010; Schäfer et al., 2010; Nevitt, 2011; Chen and Jang, 2012; Ghahremaninezhad et al., 2019). DMS also acts as a signalling molecule, for instance as a foraging cue for a variety of organisms ranging from microorganisms to marine mammals, seabirds, and crustaceans (Debose and Nevitt, 2008; Carrión et al., 2015; Mitkus et al., 2016).

The total flux of DMS to the atmosphere from the marine environment is estimated as 17.4–34.4 Tg S a^−1^ (Lana et al., 2012) representing the largest sources of atmospheric DMS. Different processes contribute to biogenic DMS formation including the cleavage of dimethylsulfoniopropionate (DMSP; Reed, 1983; Dacey and Wakeham, 1986), the degradation of other sulfonium compounds (Oduro et al., 2011; Keller et al., 2012), the biological reduction of dimethylsulfoxide (DMSO; Zinder and Brock, 1978), and the methylation of methanethiol (MT) by the methyltransferase MddA (Carrión et al., 2017). DMSP-producing phytoplankton blooms, coral reefs, and coastal saltmarshes and their vegetation are associated with high rates of DMS production. Saltmarshes are highly productive environments estimated to produce up to 1,000-fold more DMSP and DMS than the upper mixed layer of the oceans (Nedwell et al., 1994) and emit more DMS per unit area compared to the ocean (Steudler and Peterson, 1984; Yoch, 2002). In addition to the production of DMS precursors by microphytobenthos, macroalgae, and bacteria, the ecologically significant cordgrasses of the genus *Spartina/Sporobolus,* perennials that successfully colonize saltmarshes world wide (Lowe et al., 2000; Ainouche and Gray, 2016), are prolific producers of DMSP (Otte and Morris, 1994; Ansede et al., 2001; Otte et al., 2004; Bringel and Couée, 2015). In the United Kingdom, *S. anglicus* is a vigorous and successful hybrid species of *S. alterniflora* and *S. maritima* containing up to 50 μmol DMSP g^−1^ fresh weight (Otte et al., 2004). *Sporobolus anglicus* invasion in coastal saltmarshes has numerous effects on abiotic and biotic properties and the functioning of the ecosystem. Examples include the colonization of mudflats habitats that are feeding grounds for birds, as well as the replacement of native vegetation with knock-on effects on local insect populations (Wang et al., 2006).

The fact that *S. anglicus* produces large amounts of DMSP, suggests that it is likely associated with microorganisms capable of DMSP and DMS turnover and may therefore be an important hotspot for DMS cycling in saltmarshes beyond the production of DMSP. A number of DMSP-cleaving saltmarsh microorganisms have been identified previously, including bacteria (Van Der Maarel et al., 1996; Yoch, 2002; Todd et al., 2007) and saprophytic ascomycetes (Todd et al., 2009) providing a source for DMS. Degradation of DMS occurs through a number of different dissimilatory, assimilatory, and oxidative pathways, which are based on growth on DMS as a carbon and energy source, utilization of DMS as a sulfur source (reviewed in Schäfer et al., 2010). Biological oxidation of DMS is known to occur via DMS dehydrogenase (Mcdevitt, 2002; e.g., in *Rhodovulum sulfidophilum*), while trimethylamine monooxygenase has been shown to co-oxidise DMS to DMSO (Lidbury et al., 2016). Both, the degradation of DMSP and DMS, can also produce methanethiol (MeSH) as an intermediate. MeSH can be used as a sulfur source by marine bacteria through incorporation into sulfur-containing amino acids (Kiene et al., 1999; Wirth et al., 2020). Catabolism of MeSH occurs via methanethiol oxidase, which belongs to the family of selenium-binding proteins and has been purified from, e.g., *Thiobacillus thioparus* (Gould and Kanagawa, 1992) and from *Hyphomicrobium* EG (Suylen and Kuenen, 1986) and the protein-encoding gene (*mtoX*) has been identified from the DMS-degrading bacterium *Hyphomicrobium* sp. VS (Eyice et al., 2018).

In previous work, using stable isotope probing with ^13^C_2_DMS, it was shown that *Gammaproteobacteria* from the *Thiotrichales* related to *Methylophaga* assimilated DMS in pelagic samples from the English Channel (Neufeld et al., 2007b) whereas *Betaproteobacteria* related to *Methylophilaceae* and *Thiobacillus* assimilated DMS in soil and lake sediment (Eyice et al., 2015). Saltmarshes are environments with both marine and terrestrial influence and thus potentially harbor specific microbial assemblages that drive the degradation of DMS. Microbial degradation of DMS in sediment slurries of saltmarsh sediments was shown under both aerobic and anaerobic conditions (Kiene, 1988), but may also occur in close association with DMSP-producing macrophytes such as *Spartina/Sporobolus.* Here, we report on the potential to degrade DMS by microbial populations associated with *Sporobolus anglicus*.

We assessed DMSP contents of *S. anglicus,* determined potential bacterial DMS degradation in phyllosphere and rhizosphere samples, and characterised the microbial community associated with *S. anglicus* by high-throughput amplicon sequencing of 16S rRNA genes and metagenomics. Active DMS utilisers in the phyllosphere and rhizosphere were identified using ^13^C-DMS DNA stable isotope probing and the functional diversity of bacteria able to degrade methanethiol, an intermediate of DMS and DMSP metabolism was examined based on the gene encoding methanethiol oxidase (*mtoX*). Overall diversity in the phyllosphere of *S.anglicus* was assessed by high-throughput metagenomic sequencing.

## Materials and methods

### Study site, sampling and sample preparation

Whole *Sporobolus anglicus* plants including phyllosphere and rhizosphere were sampled from the Stiffkey saltmarsh (52°57′55.6″N and 0°55′24.0″E) in Norfolk (United Kingdom) in June and October 2012, in February, June, and October 2013 and in August 2014. Between 3 and 6 *S. anglicus* plants were collected from several subsites within the lower saltmarsh. These subsites were located at a maximum distance of 25 m to each other. Phyllosphere and rhizosphere samples of *S. anglicus* were used in triplicates. For the above ground parts (phyllosphere), leaf wash, leaf and stem samples were used, whereas below ground parts (rhizosphere) included root samples including endorhizosphere and rhizoplane, and rhizosphere sediment (ectorhizosphere), respectively. Leaf wash samples were prepared according to Atamna-Ismaeel et al. (2011) using 5 g of leaves and collecting the leaf surface communities (epiphytes) on a 0.22 μm Durapore® membrane filter (Merck Millipore Corporation, Darmstadt, Germany). Intact leaf and stem samples (comprising epi- and endophytes) were cut off the plants using sterile razor blades. Root samples were washed with MilliQ water to separate them from rhizosphere sediment. Loosely associated material near the roots was sampled as rhizosphere sediment. In addition to *S. anglicus* plant samples, in October 2013, bulk sediment of *S. anglicus* plants was sampled to characterise the sediment type, particle size, pH, total N, total C, organic matter, and water extractable anions (Forestry Commission Research Agency, Surrey, United Kingdom). In August 2014, *S. anglicus* phyllosphere samples were collected for metagenomics. Plants used for determination of microbial community composition and in incubation studies were generally from areas characterized by a sandy loam sediment (Table 1). An overview over the samples and experiments that were carried out can be found in Supplementary Table 1.

**Table 1:** Chemical properties of rhizosphere sediment samples from six different *Sporobolus anglicus* plants from October 2013.

								Water extractable anions				
	Textural class	pH H_2_O	Total N	Total C	C(Org)	O.M.	C:N ratio	Cl	N (NO_3_)	S (SO_4_)	P (PO_4_)	N (NO_2_)
Details	–	–	%	%	%	%	–	mg/kg	mg/kg	mg/kg	mg/kg	mg/kg
S1	Sand	7.26	0.014	0.21	0.19	0.33	15	4,292	0.33	253	<0.15	0.41
S2	Sand	7.53	0.012	0.15	0.14	0.23	13	4,874	0.36	266	<0.15	0.23
S3	Sand	7.55	0.013	0.17	0.16	0.27	13	5,633	0.56	344	<0.15	<0.05
S4	Sandy loam	7.97	0.080	1.17	1.07	1.85	15	8,762	0.80	472	<0.15	1.06
S5	Sandy loam	8.11	0.072	1.09	1.01	1.75	15	9,572	0.90	563	<0.15	0.30
S6	Sandy loam	7.93	0.097	1.44	1.31	2.26	15	9,123	1.05	581	<0.15	0.14

### DMSP and DMS measurements and DMS assimilation experiments

DMSP concentrations in phyllosphere (leaf wash, leaves, and stems) and rhizosphere (roots and sediment) samples were measured as DMS following alkaline hydrolysis as reported and described previously (Miller and Belas, 2004; Lidbury et al., 2016) in samples collected in June and October 2012 and February, June, and October 2013. In June and October 2012 biological replicates, and in February, June, and October 2013, biological and technical replicates were used. In June and October 2012 and in February and June 2013, three different plants and in October 2013, six different plants were analysed. DMS measurements of headspace gas were carried out by gas chromatography analysis using a Shimadzu GC2010plus as described by Lidbury et al. (2016). DMS consumption was assessed using approximately 0.5 g of sample which was added to a 125-ml serum vial with 25 ml marine ammonium mineral salts (MAMS) medium (Thompson et al., 1995) and sealed with a butyl rubber stopper. Initially 1 mM of DMS was added to the samples and DMS concentration in the headspace was measured over time. In order to determine endogenous DMS released from the sample material, e.g., from degradation of DMSP, DMS concentrations were also determined for sample material without addition of DMS. All DMS concentrations reported have been corrected for endogenous DMS.

### DNA extraction, high-throughput amplicon sequencing of 16S rRNA genes and metagenomics

DNA was extracted from all *S. anglicus* tissue samples, sediment samples, and stable isotope probing experiments using the FastDNA™ Spin Kit for Soil (MP Bio Science Ltd., Derby, United Kingdom) following the manufacturer’s instructions. For *S. anglicus* tissue and sediment samples, 0.5 g of sample was used. Leaf wash samples were collected on 0.22 μm filters as described above. Filters were placed in the lysis tubes and the subsequent steps performed according to the manufacturer’s instructions. DNA for the *S. anglicus* phyllosphere metagenomes was extracted from triplicate *S*. *anglicus* phyllosphere samples according to Delmotte et al. (2009). Overall, 2.4–3.0 kg leaf material was used per *S. anglicus* phyllosphere sample for the extraction of metagenomic DNA.

High-throughput amplicon sequencing was applied for taxonomic classification (16S rRNA gene) of non-enriched phyllosphere and rhizosphere tissue samples, sediment samples, “light” and “heavy” [^12^C_2_]-DMS and [^13^C_2_]-DMS incubations, and metagenomes. Amplicon samples were sequenced on the Illumina MiSeq platform, metagenomic DNA was sequenced on the HiSeq platform. Library preparation for 16S rRNA amplicon HTS was carried out according to Caporaso et al. (2012). Quantification of amplicons was carried out using the Quanti-iTTM Picogreen® dsDNA assay kit (Invitrogen Corporation) following the manufacturer’s instructions using a CFX ConnectTM Real-Time PCR Detection System (Bio-Rad Laboratories Inc., Hercules, CA, United States) to measure fluorescence. Amplicon pools (240 ng each) were cleaned up using the QIAquick PCR purification kit (QIAGEN) and their concentration and purity was determined on a QubitTM fluorometer (Invitrogen Corporation, Carlsbad, CA, United States). Amplicon pools were diluted with elution buffer (10 mM Tris-Cl) to 4 nM for sequencing on the Illumina MiSeq platform. In order to obtain enough DNA for *S. anglicus* phyllosphere metagenomic sequencing, multiple displacement amplification was carried out using the Illustra GenomiPhi V2 DNA amplification kit (GE Healthcare Life Sciences, Little Chalfont, United Kingdom) prior to sequencing. Library preparation and paired-end sequencing with a 20 pmol spike-in of PhiX (Illumina) was carried out by the Genomics facility of the School of Life Sciences at the University of Warwick.

### DNA-SIP with ^13^C-DMS

DNA-stable isotope probing (DNA-SIP) of DMS-assimilating microorganisms in phyllosphere and rhizosphere of *S. anglicus* was carried out according to Lueders et al. (2004) and Neufeld et al. (2007c). The active DMS-assimilating bacterial population in the rhizosphere sediment was investigated in June 2012 and February 2013. The active DMS-assimilating bacterial population in the phyllosphere of *S. anglicus* (leaf wash) was studied in February and June 2013. Leaf wash samples were prepared as described above. All SIP incubations were carried out in triplicates. Approximately 2 g rhizosphere sediment or 1–2 g leaf wash sample was incubated with either [^13^C_2_]-DMS or [^12^C_2_]-DMS in a 125 ml serum vial sealed with a butyl rubber stopper. Overall, 100 μmol carbon g^−1^ sample in form of either [^13^C_2_]-DMS or [^12^C_2_]-DMS was provided over three pulses. Substrate utilization was monitored using gas chromatography as described above. Samples were harvested and processed immediately after DMS was not detectable anymore.

DNA-SIP fractions were used as template for the amplification of bacterial 16S rRNA genes using the primers 341F-GC and 907R (Muyzer et al., 1993). By comparing “light” and “heavy” fractions of [^13^C_2_]-DMS and [^12^C_2_]-DMS incubations active DMS-assimilating populations were identified. Fractions were initially analysed by DGGE and subsequently subjected to 16S rRNA amplicon high-throughput sequencing. In addition, individual DGGE bands were cut out and sequenced according to Neufeld et al. (2008).

### Functional gene PCR and cloning of MTO

The presence and diversity of the gene (*mtoX*) encoding the methanethiol oxidase was assessed in leaf, root, and rhizosphere sediment samples from June 2012 and in root and rhizosphere sediment samples from October 2012 and February 2013. Functional gene PCRs were performed in a 50 μl reaction volume containing 1 × buffer including MgCl_2_, 0.8 mM dNTPs (0.2 mM each dNTP), 0.4 μM forward (MtoX41Fmodv2_inos) and reverse (MTOX346Rmod) primer (Eyice et al., 2018), 2 U Taq DNA polymerase (KAPATaq; KAPA Biosystems, Wilmington, United States), and template DNA (5–20 ng), and the reaction was made up to volume with nuclease-free sterile dH_2_O. For construction of clone libraries, DMSO (4% v/v) and BSA (0.04%, w/v) were added to enhance strand separation in GC-rich regions and to help stabilise the enzyme, respectively. PCRs were carried out in a T-100 thermocycler (Bio-Rad Laboratories Inc., Hercules, United States). PCR cycling regime and subsequent cloning of PCR products was carried out according to Eyice et al. (2018). DNA sequencing of clones was carried out by GATC Biotech GmbH (Konstanz, Germany).

### Data analysis and statistics

Raw fastq reads from 16S rRNA gene amplicon HTS were joined and demultiplexed using QIIME (Caporaso et al., 2010). Quality filtering, length truncating, and conversion of fastq to fasta was done with USEARCH v7 (Edgar, 2010). Dereplication, abundance sort and discard of singletons were also done using USEARCH v7. Operational taxonomic unit (OTU) clustering was carried out with UPARSE (Edgar, 2013), and chimeras were removed using UCHIME (Edgar et al., 2011) and the 16S rRNA gene Gold database.^1^ Taxonomy was assigned and sequences aligned with QIIME using Greengenes for reference-based assignment and alignment, respectively (Desantis et al., 2006). The alignment was filtered and a reference phylogenetic tree was made within QIIME. Rarefied OTU read abundance graphs were constructed in Microsoft Excel. Statistical analysis including non-parametric multidimensional scaling (MDS), analysis of similarity (ANOSIM) and similarity percentage analysis (SIMPER) was performed using PRIMER v6 (Clarke, 1993). In order to determine the operational taxonomic units (OTUs) most likely to explain variations between different bacterial communities and/or samples linear discriminant analysis (LDA) effect size (LEfSe) method was used (Segata et al., 2011).

Analysis of *mtoX* sequencing data was carried out using BLASTx (Altschul et al., 1990). After sequences were confirmed to be partial *mtoX* sequences they were imported into an inhouse ARB MtoX database (Eyice et al., 2018), translated, added to a seed alignment of MtoX amino acid sequences and their relatedness with other MtoX sequences was analysed using the neighbour-joining method implemented in Arb (Ludwig et al., 2004).

Metagenomics-Rapid Annotation using Subsystem Technology (MG-RAST) was used for analysis of taxonomic and functional assignment of the metagenomic reads (Meyer et al., 2008). MG-RAST was chosen for analysis of the metagenomic data due to being suggested to perform among the best compared to other metagenomic data analysis tools, such as QIIME, MEGAN, Kraken, or MethaPhlan (Lindgreen et al., 2016). Taxonomic assignment of metagenomic sequences was performed using MG-RAST and the best hit classification method against the M5NR database, which provides non-redundant integration of the GenBank (Benson et al., 2011), SEED (Overbeek et al., 2005), IMG (Markowitz et al., 2008), UniProt (Magrane and UniProt Consortium, 2011), KEGG (Kanehisa, 2002), and eggNOG (Jensen et al., 2008) database and a cut-off E-value of 1e−10. In addition, metagenomics raw reads were assembled using metaSPAdes v3.11.1 (Nurk et al., 2017). In order to compare the genetic potential for DMS utilization within the metagenomes, metagenomes were screened for functional marker genes involved in DMS utilization and subsequent degradation based on metagenomic contigs using BLAST (Altschul et al., 1990) and Prokka (Seemann, 2014). Functional marker genes used for this analysis were genes involved in DMSP demethylation (*dmdA*, *dmdB*, *dmdC*, *dmdD*), DMSP cleavage (*dddP*, *dddD*), DMS oxidation (*ddh*, *dmoA*), methanethiol degradation (*mtoX*), and genes involved in the downstream metabolism of DMSP, DMS, and methanethiol degradation products, such as genes involved in one carbon metabolism either via the tetrahydrofolate-dependent pathway (*mtdA*, *folD*, *fhs*, *fchA*, and *purU*) or the tetrahydromethanopterin-dependent pathway (*fae*, *mtdB*, *mch*, *fhcA*, and *fhcD*) and genes involved in sulfur oxidation (*pdo*, *fccB*, *cycJI*, *paps*, *ask*, *cysDN*, and *soxCDHYZ*). E-values are indicated in Supplementary Table 2. In addition, shortBRED (version 0.9.3; Kaminski et al., 2015) was used to calculate the functional marker gene abundance in the raw metagenomic reads in reads per kilo base per million mapped reads (RPKM) using reference sequences from the uniport database (Consortium, 2007) and from Prokka annotations and the uniref90 dataset was used as the reference database for shortbred_identify.py. Assessment of the metabolic pathways present in the phyllosphere samples was carried out using Prokka annotation (Seemann, 2014) and BLAST (Altschul et al., 1990) analysis at the community level and the abundance of the corresponding functional marker genes was examined using shortBRED (Kaminski et al., 2015).

### Accession numbers for datasets

16S rRNA raw read data from *Sporobolus anglicus* phyllosphere and rhizosphere samples have been submitted to the Sequence Read Archive (SRA) under the accession number PRJNA670606. Raw read data from the 16S rRNA under dimethylsulfide DNA stable isotope probing have been submitted under the accession number PRJNA670609. Read data of the three *Sporobolus anglicus* metagenomes have been submitted to the Sequence Read Archive (SRA) under the accession numbers ERX675820, ERX675821, and ERX675822. DNA sequences of methanethiol oxidase genes (*mtoX*) have been deposited in Genbank under accession numbers MW245658–MW245712.

## Results

### DMSP concentrations in *Sporobolus anglicus* tissue and sediment samples and associated DMS uptake/degradation

**Figure 1:**
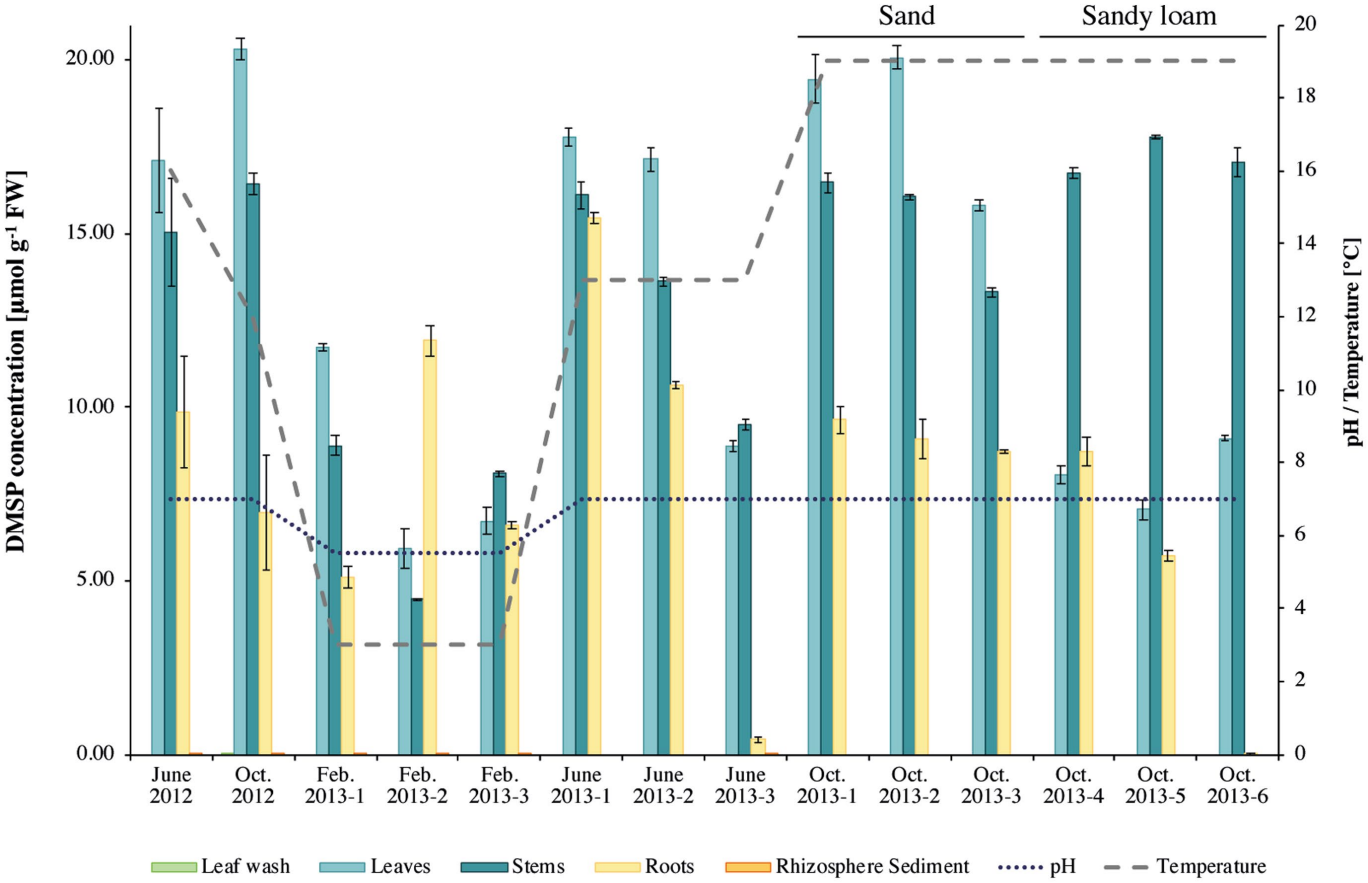
Dimethylsulfoniopropionate (DMSP) concentrations in *Sporobolus anglicus* tissue samples. DMSP concentration is given in μmol g^–1^ fresh weight of plant material for phyllosphere (leaf wash, leaf, and stem) and rhizosphere (root and sediment) samples of *Sporobolus anglicus* over an annual cycle (June 2012, October 2012, February 2013, June 2013, and October 2013). DMSP concentrations were normalized to 1 g of fresh plant material. pH and temperature at the sampling site were measured and are shown on the secondary *y*-axis. Replicate plants samples in October 2013 were from sandy sediment (as previous samples; designated October 2013–1–3, or sandy loam replicates labelled October 2013–4–6).

Analysis of the DMSP concentrations in *S. anglicus* tissue samples confirmed that plants sampled for this study had high DMSP concentrations and showed differences in DMSP concentration between plant tissues and sampling sites. The highest DMSP concentrations were detected in *S. anglicus* leaf, stem, and root tissue samples (Figure 1) with concentration up to 20 μmol g^−1^ fresh weight (FW). DMSP was not detectable or only present at very low concentrations in leaf wash and rhizosphere sediment samples. Variability in the DMSP concentration was observed, not only between different seasons and months, but also between individual plants and their tissues (Figure 1). In order to assess factors potentially contributing to the observed variation in DMSP concentration in the different *S. anglicus* tissues samples, sediment from the site of sampling of the six plants sampled in October 2013 was used for analysis of pH, water soluble anions, total nitrogen, carbon, organic carbon, organic matter (O.M.), and particle size distribution (Table 1). Sediment analysis confirmed two different soil types (sand and sandy loam) with different chemical properties (Table 1) in these six samples from October 2013.

**Figure 2:**
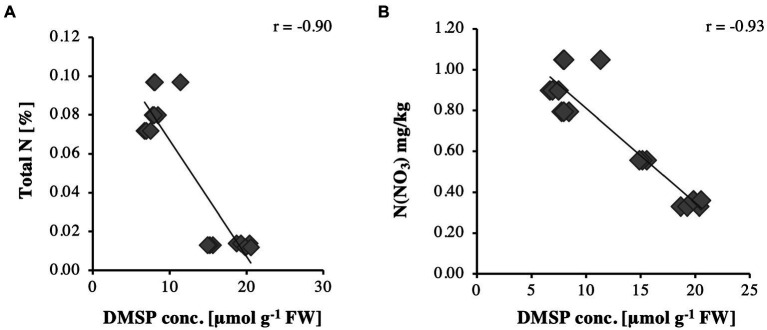
Relationships for DMSP concentrations in *Sporobolus anglicus* leaves in relation to **(A)** total nitrogen and **(B)** N(NO_3_).

The corresponding tissue DMSP concentrations (Figure 1) were highest in leaf samples of the *S. anglicus* plants growing in sand (replicate samples October 1–3, 2013), whereas the highest DMSP concentrations in the *S. anglicus* plants growing in the sandy loam were observed in the stems (Figure 1, replicate samples October 4–6, 2013). Colmer et al. (1996) showed that with increasing nitrogen concentrations DMSP concentrations in *Sporobolus* leaf blades decrease. To confirm these results with naturally grown *S. anglicus*, two scatter plots (Figure 2) were drawn for the DMSP concentrations in *S. anglicus* leaves from October 2013 (Figure 1) and the total nitrogen concentration and the nitrate concentration from NO_3_^−^ (Table 1). This showed a negative correlation between the intracellular DMSP concentrations in the leaves and the nitrogen availability in the surrounding sediment in saltmarsh grown *S. anglicus* and therefore confirms the observations made in the experimental study of *S. alterniflora* leaf blades (Figure 2).

**Figure 3:**
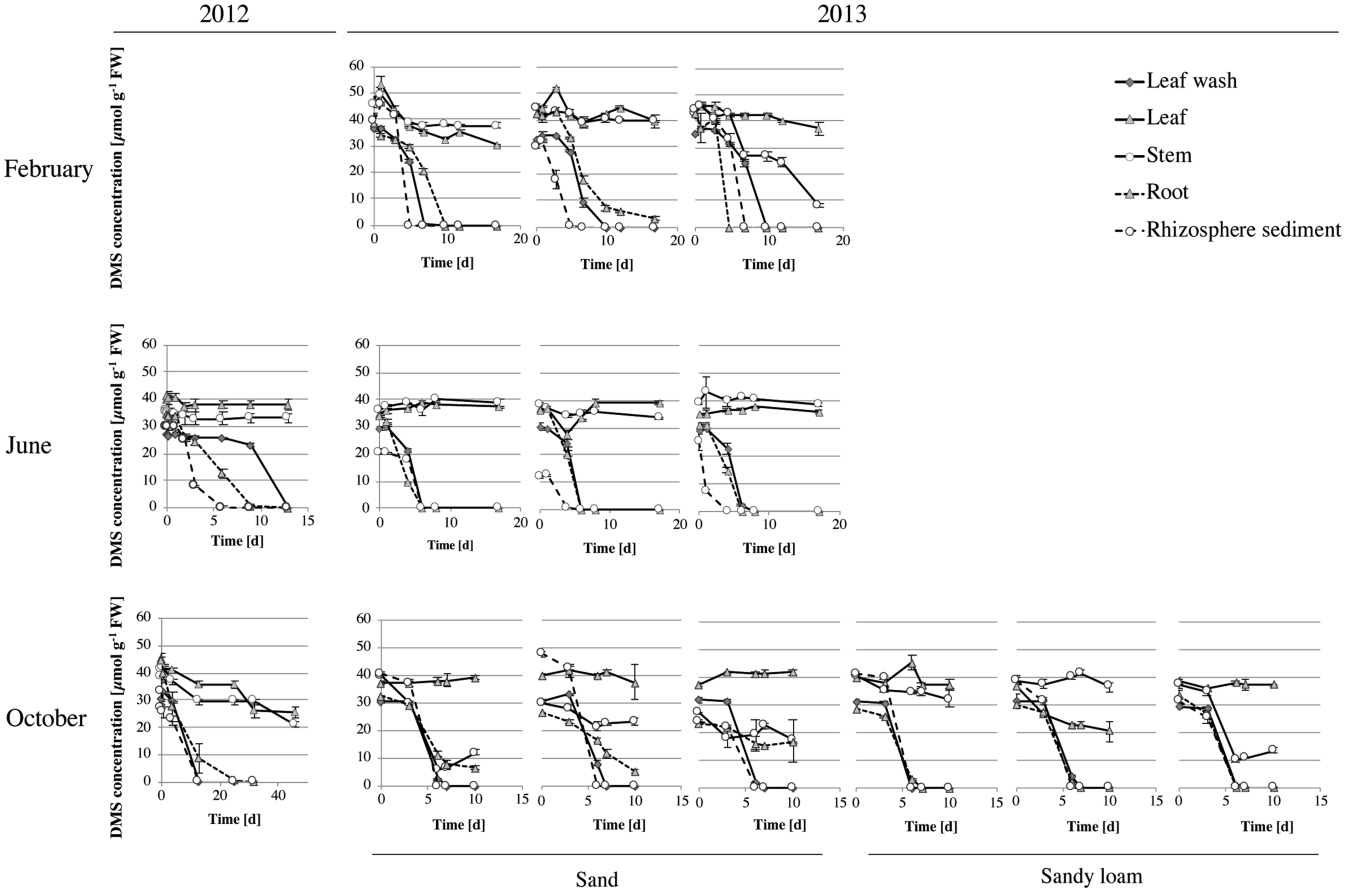
DMS degradation in *Sporobolus anglicus* tissue samples. Determination of DMS concentration was carried out with fresh leaf wash, leaf, stem, root and rhizosphere sediment samples from *S. anglicus* from June, October 2012 and February, June and October 2013. Experiments in June and October 2012 were carried out with biological replicates (three different *S. anglicus* plants) and experiments in February, June, and October 2013 were carried out with biological and technical replicates. Initially, 1 mM of DMS was added to the samples and DMS degradation was monitored over time via gas chromatography. DMS concentration is shown in μmol g^–1^ fresh weight (FW) after subtraction of background measurements. Error bars denote standard deviation (SD).

The hypothesis that *S. anglicus* is associated with DMS-degrading microorganisms was confirmed through assessment of potential DMS degradation associated with aboveground and belowground *S. anglicus* samples. All samples showed DMS degradation, with highest rates observed in leaf wash, root and rhizosphere sediment samples (Figure 3). Degradation of 1 mM DMS (0.001 mol l^−1^) in these samples typically took between 4 and 13 days, except for samples from October 2012 where the degradation of the DMS took between 13 and 25 days in leaf wash, root and rhizosphere sediment samples (Figure 3). The rhizosphere sediment samples were found to be the most active in terms of DMS uptake with rates up to 8.37 ± 0.05 μmol DMS g^−1^ sediment d^−1^ (data not shown).

### Bacterial community composition associated with *Sporobolus anglicus* and drivers of the community structure

**Figure 4:**
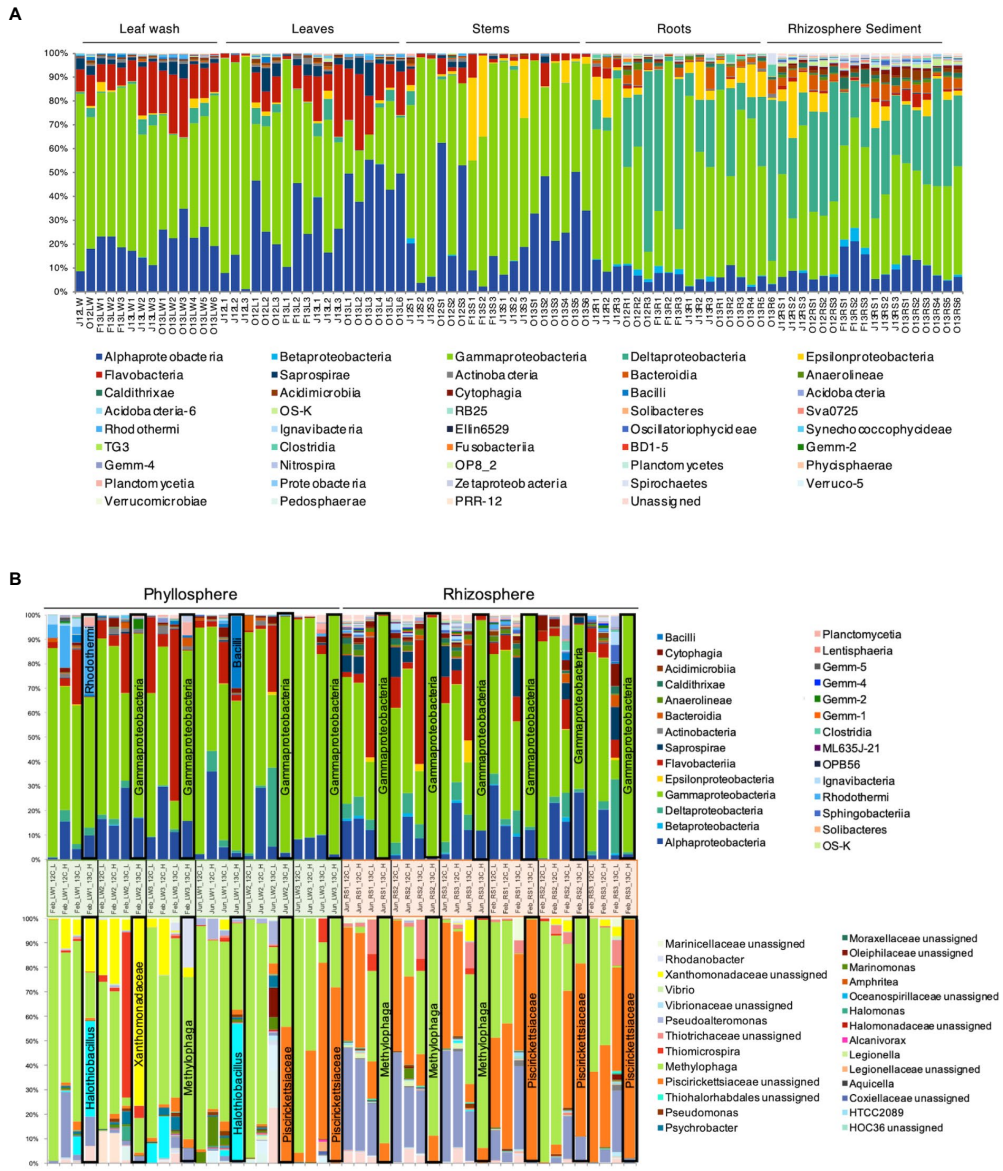
Bacterial community composition in phyllosphere and rhizosphere of *Sporobolus anglicus*. **(A)** Bacterial community composition in leaf wash (LW), leaf (L), stem (S), root (R), and rhizosphere sediment (RS) samples of *S. anglicus* in June 2012 (J12), October 2012 (O12), February 2013 (F13), June 2013 (J13), and October 2013 (O13) at class-level. Each column represents relative abundance of microbial taxa detected in a sample. **(B)** Relative distribution and phylogenetic affiliation of OTUs detected by bacterial 16S rRNA high-throughput amplicon sequencing of fractionated DNA from [^13^C_2_]-DMS and [^12^C_2_]-DMS SIP incubations of phyllosphere (leaf washings—LW) and rhizosphere samples (sediment—RS) of *S. anglicus* from June and February at class-level (top) and genus-level (bottom, most abundant genera) after incorporation of 100 μmol C g^–1^ sample. Each column represents relative abundance of microbial taxa detected in a sample. Columns marked with a black frame are ^13^C-“heavy” fractions that should represent the enriched community under [^13^C_2_]-DMS treatment and therefore the active DMS-degrading community.

High-throughput amplicon sequencing of the 16S rRNA gene provided a detailed analysis of the bacterial community associated with *S. anglicus* phyllosphere and rhizosphere samples from June and October 2012 and February, June and October 2013. The bacterial community composition of *S. anglicus* (Figure 4A; Table 2) demonstrated differences in the major bacterial classes in phyllosphere compared to rhizosphere samples based on relative abundances. In phyllosphere samples (leaf wash, leaves, and stem) abundant bacterial classes were *Alphaproteobacteria* and *Gammaproteobacteria* (Figure 4A; Table 2). In addition, *Flavobacteria* were abundant in leaf wash and leaf samples. *Deltaproteobacteria* were found less abundant in the phyllosphere samples (Table 2). *Gammaproteobacteria* were the most abundant bacterial class associated with *S. anglicus* rhizosphere samples (root and sediment). Also, *Deltaproteobacteria* showed a high abundance in the rhizosphere (Figure 4A; Table 2). *Alphaproteobacteria*, *Flavobacteria* and *Saprospirae* were less abundant in rhizosphere samples compared to phyllosphere samples (Table 2). The bacterial community composition in the phyllosphere and rhizosphere samples of *S. anglicus* was thus different.

**Table 2:** Relative Abundance (±SD) of microbial classes in phyllosphere and rhizosphere samples of high-throughput amplicon sequencing of the 16S rRNA gene.

Sample microbial class	Phyllosphere			Rhizosphere	
	Leaf wash	Leaf	Stem	Root	Sediment
Alpharoteobacteria	20.5% ± 6.5%	31.50%	24.3% ± 18%	6.90%	9.80%
Gammaproteobacteria	55.4% ± 11.5%	46%	63.2% ± 19.6%	50.80%	37.50%
Deltaproteobacteria	2.30%	2.30%	0.56%	27.60%	31%
*Flavobacteria*	14.7% ± 5.9%	12.70%			
*Saprospirae*	2.9% ± 1.6%	3.70%			

**Figure 5:**
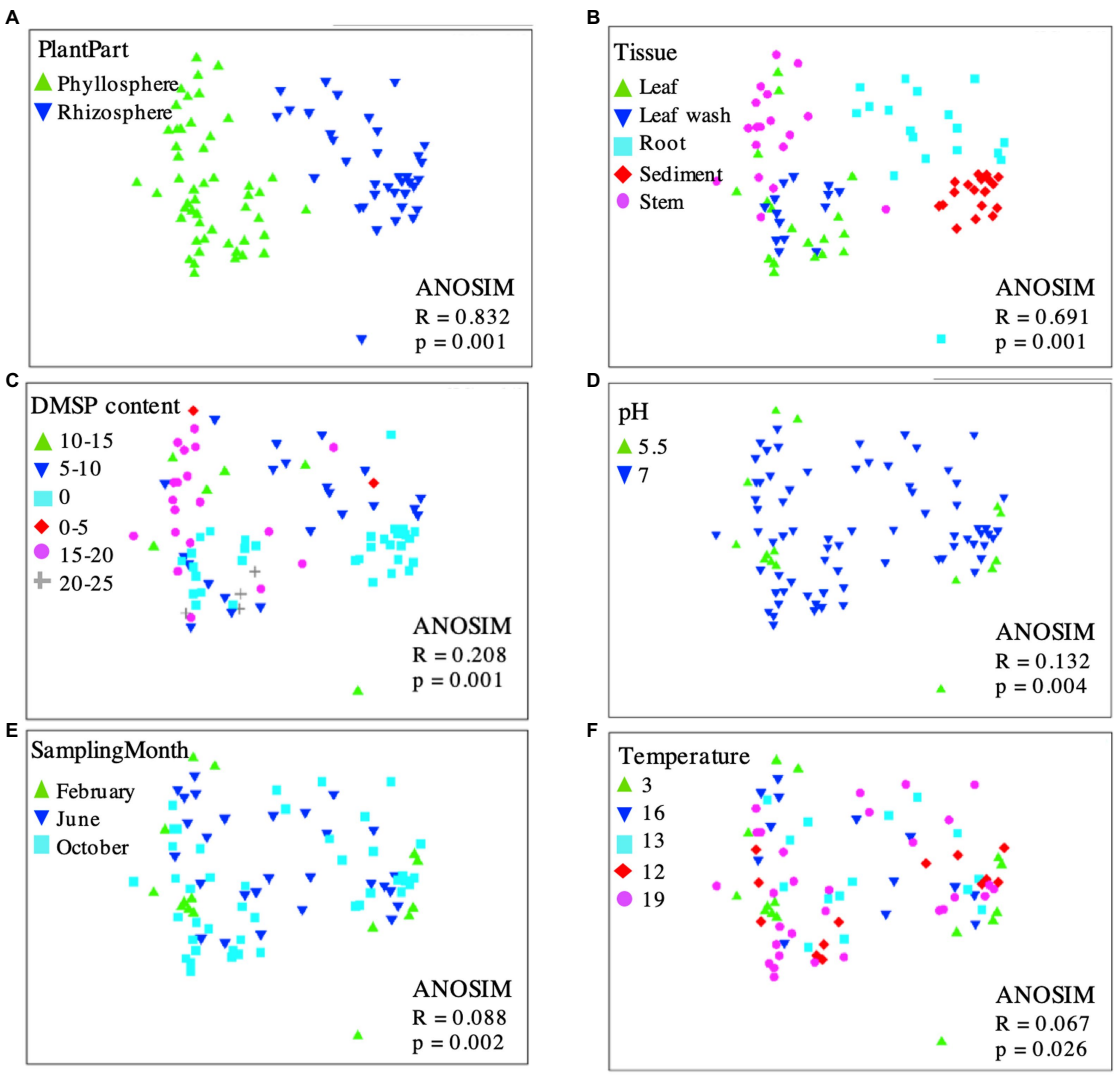
Multidimensional scaling (MDS) plot displaying 16S rRNA gene-based bacterial community composition at bacterial genus level of leaf wash, leaf, stem, root and sediment samples with superimposed codes representing plant part **(A)**, tissue sample **(B)**, DMSP content **(C)**, pH **(D)**, sampling month **(E)**, or temperature **(F)**. Proximity of points reflects similarity. OTUs diversity among phyllosphere and rhizosphere samples is highly similar, indicating bacterial communities are independent of DMSP content, pH, sampling month and temperature but not tissue or plant part. MDS ordinates and ANOSIM (analysis of similarity) were determined by calculating a dissimilarity matrix (Bray-Curtis) based on the OTUs. Calculations, MDS, and ANOSIM were done using PRIMER v6.

The linear discriminant analysis (LDA) effect size (LEfSe) method (Segata et al., 2011) confirmed the differences between phyllosphere and rhizosphere microbial diversity and identified the OTUs that differ in phyllosphere and rhizosphere samples (Supplementary Figure 1; Figure 5). The OTUs which were most dominant in rhizosphere samples compared to phyllosphere samples were *Desulfobacterales*, *Desulfobulbacaea*, *Chromatiales*, and *Thiotrichales*. The most differentially abundant bacterial taxa in phyllosphere samples were *Oceanospirillales*, *Pseudomonadales*, *Psychrobacter*, and *Rhodobacterales*.

To assess which factors might affect the community composition associated with *S. anglicus,* multidimensional scaling (MDS) was performed. Plots displaying the 16S rRNA gene-based bacterial community composition at the genus level of all phyllosphere and rhizosphere samples were calculated, showing data points with superimposed codes representing plant part, sample tissue, DMSP content, pH, sampling month, and temperature (Figure 5). Overall, phyllosphere and rhizosphere microbial communities separated in MDS analysis according to sample type (Figure 5A) and sampled tissue/location (Figure 5B), but there was no evident separation according to DMSP content (Figure 5C), pH (Figure 5D), sampling month (Figure 5E), or temperature (Figure 5F).

Operational taxonomic units primarily providing the discrimination between the two observed sample clusters (phyllosphere and rhizosphere; Figure 5A) were identified using SIMPER analysis. The OTUs contributing most to the observed community differences were *Marinomonas* (22.27%, order *Oceanospirillales*), *Rhodobacteraceae* (18.73%, order *Rhodobacterales*), and *Psychrobacter* (12.55%, order *Pseudomonadales*) within the phyllosphere, and *Chromatiales* (14.04%), *Desulfobulbaceae* (13.31%, order *Desulfobacterales*) and *Desulfobacteraceae* (8.89%, order *Desulfobacterales*) in the rhizosphere. These analyses corresponded well to the results obtained by the LEfSe analysis.

### Active dimethylsulfide-degrading bacteria identified by ^13^C-DMS DNA-SIP

To identify active DMS-degrading microorganisms, DNA-SIP incubations were carried out with rhizosphere sediment from June 2012 and February 2013 and phyllosphere samples of *S. anglicus* (leaf wash) from February and June 2013. DNA was extracted from phyllosphere and rhizosphere SIP incubations with ^13^C-labelled DMS (and control incubations with ^12^C-DMS) after degradation of approximately 100 μmol carbon g^−1^ sample. Initial evaluation of SIP fractions by DGGE analysis of bacterial 16S rRNA amplicons indicated successful labelling (data not shown). Bacterial 16S rRNA amplicon high-throughput sequencing was done for the “heavy” and “light” ^12^C- and ^13^C-labelled fractions of each experiment. A total of 6,636,546 quality-filtered, chimera-checked sequences were obtained from 48 samples and assigned to 1,115 distinct OTUs at 97% identity (average 138,261 ± 46,868 reads per sample). Taxonomic profiling showed distinct community profiles in “heavy” and “light” DNA fractions of phyllosphere and rhizosphere SIP samples (Figure 4B). Changes in the read abundance for certain OTUs in distinct DNA fractions identified bacterial populations assimilating the carbon of the labelled DMS. The ^13^C-heavy DNA fractions of rhizosphere samples were greatly enriched in *Gammaproteobacteria* and, more specifically, by *Methylophaga* (family *Piscirickettsiacaea*, order *Thiotrichales*) and other (unassigned) *Piscirickettsiacaea* (Figure 4B). ^13^C-heavy DNA fractions from the phyllosphere were enriched in *Gammaproteobacteria* [including *Methylophaga* and other (unassigned) *Piscirickettsiacaea*] but also highlighted a range of other genera as DMS degraders (Figure 4B) with more variation between seasons and plants than observed with the rhizosphere samples. In February, the OTUs identified in the ^13^C-‘heavy’ fraction were *Halothiobacillus*, *Xanthomonadaceae*, *Methylophaga*, and *Rhodanobacter*. In June, *Halothiobacillus* was also identified in the ^13^C-“heavy” fraction as well as *Alicyclobacillaceae* and *Piscirickettsiacaea*. *Halothiobacillus* (*Gammaproteobacteria*) oxidizes inorganic sulfur compounds such as thiosulfate, S and sulfides and hydrogen sulfide to sulfate (Kelly and Wood, 2000), but growth on DMS has not previously been reported. The family *Xanthomonadaceae* also belongs to the class *Gammaproteobacteria* and includes species that are plant-associated including plant pathogens. *Rhodanobacter* belongs to the family *Xanthomonadaceae* and marine isolates closely related to *Rhodanobacter* have been shown to grow on DMS as sole carbon source (Alvarez et al., 2009). In the ^13^C-“heavy” fraction in the phyllosphere *Alicyclobacillaceae* were also identified by HTS and *Alicyclobacillus disulfidooxidans* was identified by sequencing of DGGE bands in the ^13^C-“heavy” fraction. *Alicyclobacillaceae* belong to the class *Bacilli* and *Alicyclobacillus disulfidooxidans* is a disulphide-oxidizing bacterium that was shown to grow on organic substrates like glucose, but also on sulfur substrates such as cysteine (Dufresne et al., 1996). However, it has not been shown if this microorganism can degrade DMS. The similarity seen between some ^12^C control and ^13^C incubations is likely a consequence of enrichment of the same organisms occurring in the ^12^C control incubations, but to an extent that is strong enough to effect a change in the community DNA which becomes sufficiently dominated by the relevant organisms. It has previously been demonstrated that small amounts of ^12^C DNA smear along the CsCl gradient leading to that background signal being picked up from fractions that would normally show the diversity of the heavy DNA fraction; however if ^13^C DNA is absent, these small amounts of ^12^C DNA can lead to that background signal becoming visible (Neufeld et al., 2007a).

Thus, SIP identified several candidate DMS-degrading populations including some that were previously linked to DMS cycling (*Methylophaga* and other *Piscirickettsiacaea*), as well as taxa not previously shown to degrade DMS, but which are shown here as having the potential to contribute to DMS degradation in the phyllosphere of *S. anglicus*.

At the genus level, *Methylophaga* (family *Piscirickettsiacaea*), *Halothiobacillus* (family *Halothiobacillaceae*), or *Rhodanobacter* (family *Rhodanobacteraceae*) were not detected in the 16S rRNA gene sequencing data of the same unenriched *S. anglicus* samples, suggesting that these genera were present at a low abundance. Also, the families *Halothiobacillaceae*, *Rhodanobacteraceae*, *Xanthomonadaceae*, and *Alicyclobacillaceae* were not detectable in the 16S rRNA gene sequencing data of the same unenriched *S. anglicus* samples. However, the family *Piscirickettsiacaea* (family of *Methylophaga*) could be detected in *S. anglicus* unenriched samples with relative abundances ranging from 0.1% in some leaf wash samples to 39.2% in root samples and 37.6% in rhizosphere sediment samples (data not shown) highlighting these as potentially abundant DMS degrading bacteria in association with *S. anglicus* especially below ground.

### Functional diversity of the *mtoX* gene

**Figure 6:**
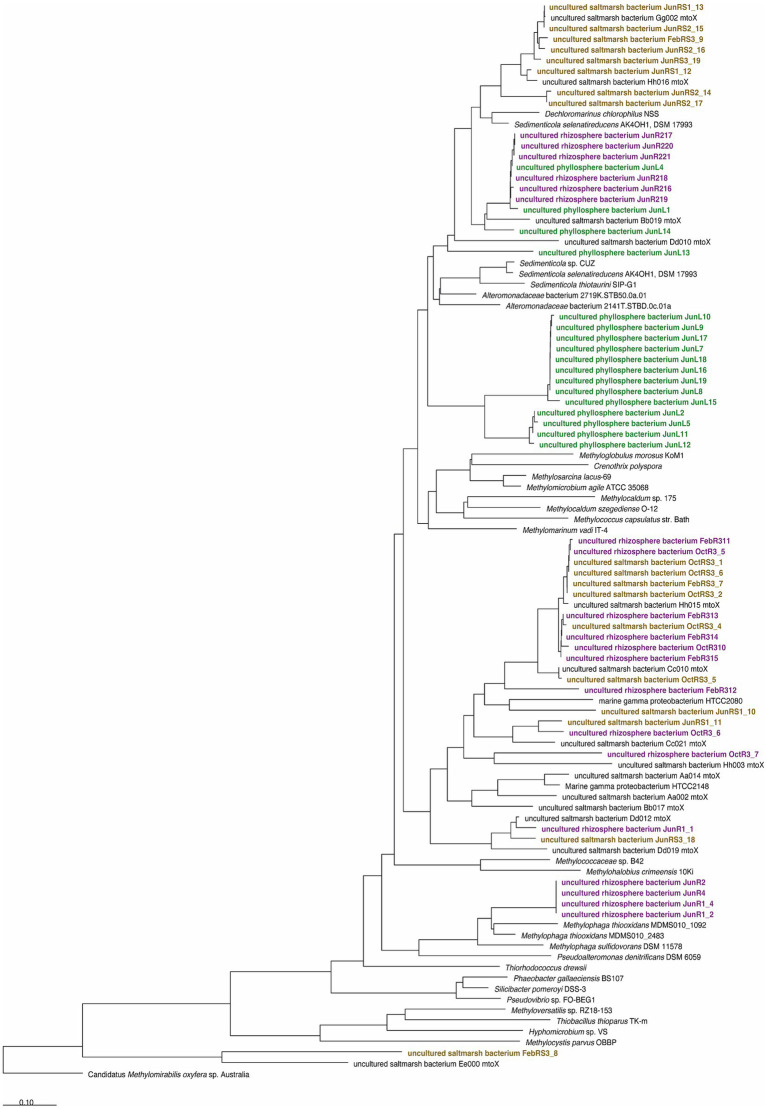
Diversity of methanethiol oxidase containing microorganisms associated with *Sporobolus anglicus* based on analysis of translated amino acid sequences derived from cloned *mtoX* amplicons. Sequence labels are coded according to time of sampling (February 2012; June and October 2013, respectively) and origin of sequences (RS, rhizosphere sediment, brown; R, root, purple; and L, leaf, green). The tree was obtained by Neighbour-Joining analysis in ARB based on an amino acid sequence alignment and the use the MtoX sequence of Hyphomicrobium sp. VS as a filter. A distance correction using the PAM matrix was used as implemented in ARB. The final dataset contained 204 valid alignment columns (positions 98–301 of the *Hyphomicrobium* sp. VS MtoX sequence).

The *mtoX* gene encoding the methanethiol monooxygenase was used as a functional marker gene to detect methanethiol-degrading bacteria (Eyice et al., 2018). This may include DMS-degrading microorganisms that degrade DMS via methanethiol such as *Methylophaga* species (Boden et al., 2010; Kröber and Schäfer, 2019), and DMSP-degrading bacteria that may degrade MeSH generated through the DMSP demethylation pathway such as *Ruegeria pomeroyi* (Reisch et al., 2008; Eyice et al., 2018). *mtoX* gene amplification was attempted on leaf, root, and sediment samples from June 2012 and with root and sediment samples from October 2012 and February 2013. The *mtoX* gene was successfully amplified from all samples demonstrating that *S. anglicus* is colonised by methanethiol-degrading microorganisms. With the exception of a clade of sequences that was detected in the phyllosphere only, most of the *mtoX* sequences obtained from phyllosphere (marked in green) and rhizosphere samples (marked in red and brown) clustered with *mtoX* clones previously reported from surface sediment of the same saltmarsh (Figure 6). None of the sequences clustered particularly closely with *mtoX* from bacterial isolates, but the closest cultivated relatives of most *S. anglicus* associated *mtoX* were from *Gammaproteobacteria*, including *Sedimenticola* spp., marine *Gammaproteobacteria*, *Methylococcaceae*, and *Methylophaga* spp. (Figure 6). It is therefore likely that these *mtoX* were from unidentified *Gammaproteobacteria*, and that these represent some of the gammaproteobacterial taxa identified as active DMS degraders by SIP. In the future, this could be investigated by metagenomic sequencing of SIP fractions and isolation of yet unidentified DMS degraders from these environments.

### Microbial diversity and functional potential of *Sporobolus anglicus* phyllosphere associated microorganisms assessed via metagenomics

Direct metagenomic analysis based on sequencing of phyllosphere community DNA of three *S. anglicus* phyllosphere samples was carried out after amplification of DNA by MDA. It should be noted, that genomic amplification with, e.g., GenomiPhi can introduce bias into the sequencing library (Ballantyne et al., 2007; Yilmaz et al., 2010). Similar to 16S rRNA amplicon-based diversity analysis, the taxonomic assignment of metagenomic reads showed that they were dominated by *Proteobacteria* and more specifically by *Gammaproteobacteria* and *Alphaproteobacteria*, but other phyla like *Chloroflexi* and *Firmicutes* were also abundant (Supplementary material 1; Figure 1A). The microbial families *Halothiobacillaceae*, *Rhodanobacteraceae*, *Xanthomonadaceae*, and *Alicyclobacillaceae* detected during DMS-SIP incubations via 16S rRNA HTS were not abundant in *S. anglicus* phyllosphere metagenomes. However, *Methylophaga* (family *Piscirickettsiacaea*) was found in 0.1–0.2% of the metagenomic reads of the *S. anglicus* phyllosphere.

A comparison of the taxonomic diversity to phyllosphere metagenomes of *Arabidopsis thaliana*, clover, soybean, and rice showed major differences in community composition between those plants and *S. anglicus* likely reflecting the differences in habitat (see Supplementary material 1).

### Metabolic pathways of organosulfur compound degradation

**Figure 7:**
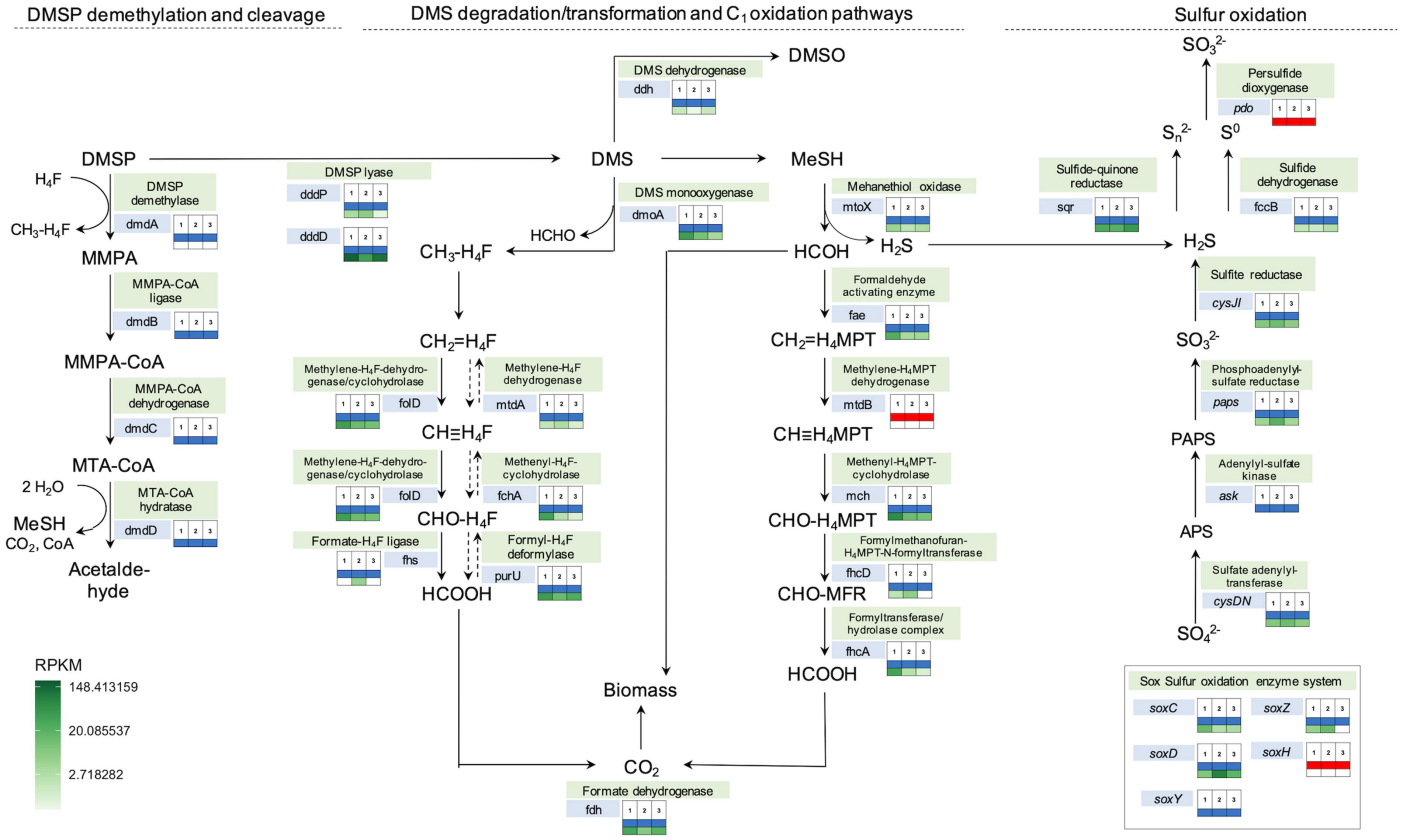
Metabolic pathways involved in DMS degradation/transformation, one-carbon and sulfur oxidation annotated with presence/absence and abundance (RPKM) of specific genes in the three *Sporobolus anglicus* phyllosphere metagenomes. The analysis is based on Prokka annotations, tBLASTn, BLASTx, and shortBRED gene searches among the three different *S. anglicus* phyllosphere metagenomes (1–3). The colour-coded boxes next to the genes indicate the presence (blue) or absence (red) of a gene in each metagenome inferred by BLAST searches (second table row) and the abundance of these genes in *Sporobolus* metagenomic reads in reads per kilo base per million mapped reads (RPKM) shown as heatmap colours (third table row). Exact RPKM values and explanation of abbreviations can be found in Supplementary Table 2. DMSP, dimethylsulfoniopropionate; DMS, dimethylsulfide; MMPA-3, methiolpropionate; MTA, 5’-Desoxy-5′-methylthioadenosin; HCHO, formaldehyde; APS, adenosine phosphosulfate; and PAPS, phosphoadenosine 5′-phosphosulfate.

Assessment of the metabolic pathways present in the phyllosphere samples demonstrated the presence of various enzyme encoding genes of pathways relevant for the degradation of DMSP, DMS and methanethiol, as well as for assimilation of C1 units from DMS into biomass and the degradation of sulfide (Figure 7). Important genes for the identification of possible routes of DMSP and DMS metabolism are genes encoding proteins involved in DMSP cleavage (DMSP lyases—*dddP* and *dddD*), DMS oxidation (DMS dehydrogenase—*ddh*, DMS monooxygenase—*dmoA*), and methanethiol degradation (Methanethiol oxidase—*mtoX*), which were all present in the investigated metagenomes (Figure 7). The metabolic potential detected in incubation studies and SIP experiments was thus reflected in detection of functions that would be expected to produce and degrade DMS in the phyllosphere.

The phylogenetic affiliation of the organisms encoding these genes was inferred by blast searches of the relevant genes detected in the metagenomic contigs against the NCBI nr database (Supplementary Table 2). This showed that taxa involved in DMS degradation (represented by closest blast hits to the genes *dmoA*, *ddh* and *mtoX*) were *Gammaproteobacteria* and *Alphaproteobacteria* (class *Rhodobacterales*). The abundance of the gene encoding the DMS monooxygenase (*dmoA*) was higher in the *Sporobolus anglicus* phyllosphere metagenomes compared to the DMS dehydrogenase (*ddh*), suggesting that DMS is more likely to be degraded to methanethiol and used as a carbon and sulfur source via its subsequent degradation to formaldehyde and hydrogen sulfide via the methanethiol oxidase (*mtoX*) than the oxidation to DMSO via the DMS dehydrogenase (Figure 7). DMS is also known to be degraded to methanethiol via a methyltransferase (e.g., in *Methylophaga thiooxydans*; Boden et al., 2010) but the gene encoding this enzyme remains unidentified and therefore its presence in the metagenome cannot be assessed. Potential microbial classes involved in sulfur metabolism were mainly *Alphaproteobacteria* and some *Gammaproteobacteria* (Supplementary Table 2). Although not detectable at the genus level in the 16S rRNA data, *Methylophaga* sp. was an abundant close hit for several genes involved in C_1_ metabolism (marked in yellow), e.g., *mch* (Methenyl-H_4_MPT-cyclohydrolase), *fhcA* (Formyltransferase/−hydrolase), *foldD* (Methylene-H_4_F-dehydrogenase/cyclohydrolase), *mtdA* (Methylene-H_4_F dehydrogenase), and *purU* (Formyl-H_4_F-deformylase; Supplementary Table 2) strengthening the importance of *Methylophaga* (*Piscirickettsiacaea*) in C_1_ and DMS cycling.

## Discussion

### Saltmarsh grown *Sporobolus anglicus* DMSP concentrations are affected by sediment type but not by season

Although the observation that *S. anglicus* contains DMSP is not a novel finding, it was important in the context of our analyses to assess the environmental and seasonal factors that could affect the DMSP content of *S. anglicus* sampled in a natural saltmarsh. The DMSP concentrations that we measured were similar to those reported previously (Van Diggelen et al., 1986; Mulholland and Otte, 2000, 2002), but our measurements suggested that plants sampled in a saltmarsh in different seasons may only have minor variation in DMSP content over the annual cycle. The low or undetectable levels of DMSP in leaf wash and rhizosphere sediment samples (detection threshold approx. 1 μM) is likely due to the intracellular storage of DMSP in *S. anglicus* (Van Diggelen et al., 1986; Otte et al., 2004; Husband et al., 2012). Microbial degradation of DMSP released from plants is likely to keep DMSP concentration low in the surrounding environment. Sediment properties affected the DMSP content in different tissues of *Sporobolus* plants as shown in previous studies with greenhouse or laboratory grown *Sporobolus.* Here we show that plants sampled from locations with different sediment properties (sand or sandy loam) had different tissue DMSP concentrations. As expected, a higher total N and total C content was shown for the sandy loam in this study (Table 1) reemphasising that soil type and texture are important factors influencing the distribution of these compounds including nitrogen (Powell Gaines and Gaines, 1994; Bechtold and Naiman, 2006). Elevated nitrogen concentrations in the sediment correlated with decreasing DMSP concentrations in *S.anglicus* (Figure 2; Table 1) as shown previously (Otte and Morris, 1994; Colmer et al., 1996; Mulholland and Otte, 2000). Similar correlation between nitrogen and DMSP has been shown in *Wollastonia biflora* (Hanson et al., 1994), and phytoplankton (Turner et al., 1988; Groene and Kirst, 1992; Keller and Korjeff-Bellows, 1996).

### *Sporobolus anglicus* is colonised by DMS-degrading bacteria in the phyllosphere and rhizosphere

The extent to which the *Sporobolus* phyllosphere is a habitat for DMS-degrading bacteria had not been explored previously. Other volatile compounds such as methanol and methyl chloride have been shown to be utilised by phyllosphere microorganisms previously (Sy et al., 2005; Knief et al., 2010; Nadalig et al., 2011) it thus seemed likely that DMS could also be a substrate for phyllosphere bacteria (Schäfer et al., 2010). This work confirmed that DMS degrading bacteria occur in the phyllosphere, experiments showed similar potential rates of DMS degradation by leaf wash and rhizosphere samples. DMS cycling in saltmarshes may therefore not be limited to sediment microbial communities (Kiene, 1988; Ansede et al., 2001), but also include aboveground phyllosphere communities. This previously unrealised aspect of DMS cycling in coastal environments calls for further analyses of DMS cycling above and below ground, respectively.

### Distinct active DMS degraders inhabit the *Sporobolus anglicus* phyllosphere and rhizosphere

Active DMS degraders in the rhizosphere were predominantly *Piscirickettsiacaea*, *Methylophaga* spp. and related microorganisms, which have previously been implicated in DMS degradation, either based on isolated strains (Zwart De et al., 1996; Janvier et al., 2003, Schäfer, 2007) or finding of *Methylophaga* and related bacteria in DMS-enriched cultures of marine samples and in SIP experiments (Vila-Costa et al., 2006; Neufeld et al., 2007b). Interestingly, these *Gammaproteobacteria* also potentially contribute to DMS cycling in the rhizosphere and phyllosphere of *Sporobolus anglicus* plants. Some rhizosphere samples contained abundant populations of *Piscirickettsiaceae* as shown by 16S rRNA amplicon sequencing of unenriched samples. SIP identified a wider range of DMS-degrading bacterial populations in the phyllosphere, including *Halothiobacillus* and *Xanthomonadaceae*. In the phyllosphere, the dominant OTUs differed not only between seasons (February and June) but also between plants suggesting a patchier distribution of these potential DMS degraders seasonally and spatially, which may indicate a lower overall abundance of these bacteria.

### *mtoX* in the phyllosphere and rhizosphere of *Sporobolus anglicus* supports presence of DMSP and DMS degrading microorganisms

The detection of the gene encoding the methanethiol oxidase, *mtoX,* in unenriched microbial communities demonstrated that MT-degrading microorganisms are closely associated with *S. anglicus.* Sequences of *mtoX* clustering closely with *Methylophaga* are likely to originate from uncultivated *Piscirickettsiacaea* shown to be involved in DMS degradation by SIP. The finding of an *mtoX* clade that was only detected in the phyllosphere suggests that some of these *Gammaproteobacteria* may have a habitat preference, but a better taxonomic placement of these sequences would require more detailed analysis of neighbouring genes in their genomic context or resolving the corresponding genomes as metagenome assembled genomes.

The application of different approaches to characterise *S. anglicus*-associated microorganisms degrading DMS and related sulfur compounds provided new insights. Although it is challenging to integrate these different datasets seamlessly, the following conclusions can be drawn. Based on SIP, the potential for DMS cycling by *Piscirickettsiaceae* is clear, but other DMS degraders outside this family were also identified in phyllosphere samples. SIP incubations may favour fast-growing organisms, which potentially outcompete slower growing ones that may be more dominant *in situ*. Therefore identification by SIP is not necessarily a reflection of their dominance or contribution to the process. This issue could be addressed in future studies under substrate-limiting conditions, ideally using small-scale chemostat experiments.

Unsurprisingly, not all OTUs identified based on SIP were found based by 16S rRNA sequencing in unenriched samples, however, *Piscirickettsiaceae* were present at up to 39% read abundance in some unenriched rhizosphere samples clearly demonstrating that these bacteria can be abundant members of the community. They are thus likely relevant for cycling of DMS (and other C1 compounds) *in situ*. This was further supported by finding several hits to C1 cycling genes in the metagenomes which were affiliated with *Methylophaga* and related organisms and *mtoX* amplicons affiliated with this group. Other candidate DMS degraders may be less abundant *in situ*, and the lack of genes of sulfur and C1 cycling matching such taxa may be due to a lack of genomes of representative DMS-degrading strains in the sequence databases. This can be illustrated using *Methylophaga* as an example. DMS metabolism has only been reported for three *Methylophaga* species and only in genomes of two of these, *M. thiooxydans* and *M. sulfidovorans*, have genes involved in DMS metabolism been identified (Kröber and Schäfer, 2019). Yet all known *Methylophaga* genomes identify these organisms as methylotrophs growing on a range of C1 compounds, but non-DMS degrading members lack diagnostic genes for organosulfur metabolism such as *mtoX*. As yet uncultivated DMS-degrading representatives of other taxa may either have distinct metabolic pathways of DMS degradation or the fact that reference genomes lack diagnostic genes such as *mtoX* makes it impossible at present to link their potential activity based on functional genes and identity according to the 16S rRNA gene.

## Conclusion

*S. anglicus* and related species of cordgrasses represent a significant component of saltmarsh plant communities world-wide, some of these are invasive, influencing the ecology and development of coastal marine environments. Although the role in production of DMSP by several *Sporobolus* species previously known as *Spartina* has been realised, their role in hosting microbial communities contributing to the cycling of DMS had not been explored in detail. The diversity of DMS-degrading microorganisms associated with *S. anglicus* was greater than previously realised and the observation of considerable potential of above-ground, plant-associated DMS degradation demonstrates that DMS cycling in coastal ecosystems is more complex than previously appreciated.

## Data availability statement

The datasets presented in this study can be found in online repositories. The names of the repository/repositories and accession number(s) can be found at: https://www.ncbi.nlm.nih.gov/search/all/?term=PRJNA670606, PRJNA670606, https://www.ncbi.nlm.nih.gov/search/all/?term=PRJNA670609, PRJNA670609, https://www.ncbi.nlm.nih.gov/search/all/?term=ERX675820, ERX675820, https://www.ncbi.nlm.nih.gov/search/all/?term=ERX675821, ERX 675821, https://www.ncbi.nlm.nih.gov/search/all/?term=ERX675822, ERX675822, https://www.ncbi.nlm.nih.gov/genbank/, MW245658, MW245659, MW245660, MW245661, MW245662, MW245663, MW245664, MW245665, MW245666, MW245667, MW245668, MW245669, MW245670, MW245671, MW245672, MW245673, MW245674, MW245675, MW245676, MW245677, MW245678, MW245679, MW245680, MW245681, MW245682, MW245683, MW245684, MW245685, MW245686, MW245687, MW245688, MW245689, MW245690, MW245691, MW245692, MW245693, MW245694, MW245695, MW245696, MW245697, MW245698, MW245699, MW245700, MW245701, MW245702, MW245703, MW245704, MW245705, MW245706, MW245707, MW245708, MW245709, MW245710, MW245711, and MW245712.

## Author contributions

EK and HS conceived the research, analyzed the data, and wrote the manuscript. EK carried out the experimental research. AM supported the data analysis. All authors contributed to the article and approved the submitted version.

## Funding

This study was supported with funding from the Natural Environment Research Council (NERC; grants NE/E013333/1 and NE/H008918/1).

## Conflict of interest

The authors declare that the research was conducted in the absence of any commercial or financial relationships that could be construed as a potential conflict of interest.

## Publisher’s note

All claims expressed in this article are solely those of the authors and do not necessarily represent those of their affiliated organizations, or those of the publisher, the editors and the reviewers. Any product that may be evaluated in this article, or claim that may be made by its manufacturer, is not guaranteed or endorsed by the publisher.

## Footnotes

^1^ http://drive5.com/uchime/gold.fa
